# Anti-Inflammatory Potential of n-3 Polyunsaturated Fatty Acids Enriched Hen Eggs Consumption in Improving Microvascular Endothelial Function of Healthy Individuals—Clinical Trial

**DOI:** 10.3390/ijms21114149

**Published:** 2020-06-10

**Authors:** Ana Stupin, Martina Mihalj, Nikolina Kolobarić, Petar Šušnjara, Luka Kolar, Zrinka Mihaljević, Anita Matić, Marko Stupin, Ivana Jukić, Zlata Kralik, Manuela Grčević, Gordana Kralik, Vatroslav Šerić, Ines Drenjančević

**Affiliations:** 1 Department of Physiology and Immunology, Faculty of Medicine Osijek, Josip Juraj Strossmayer University of Osijek, J. Huttlera 4, HR-31000 Osijek, Croatia; ana.stupin@mefos.hr (A.S.); martina.mihalj@gmail.com (M.M.); nikolina.bilic.dujmusic@gmail.com (N.K.); psusnjara1@gmail.com (P.Š.); lukakolar.vu@gmail.com (L.K.); zrinka.mihaljevic@mefos.hr (Z.M.); anita.matic@mefos.hr (A.M.); marko.stupin@gmail.com (M.S.); ivana.jukic@mefos.hr (I.J.); 2 Scientific Center of Excellence for Personalized Health Care, Josip Juraj Strossmayer University of Osijek, Trg Svetog Trojstva 3, Hr-31000 Osijek, Croatia; zlata.kralik@fazos.hr (Z.K.); mgrcevic@pfos.hr (M.G.); gkralik@fazos.hr (G.K.); 3 Department of Pathophysiology, Physiology and Immunology, Faculty of Dental Medicine and Health Osijek, Josip Juraj Strossmayer University of Osijek, Cara Hadrijana 10E, HR-31000 Osijek, Croatia; 4 Department of Dermatology and Venereology, Osijek University Hospital, J. Huttlera 4, HR-31000 Osijek, Croatia; 5 Department of Internal Medicine, Vukovar General Hospital, Županijska ulica 35, HR-32000 Vukovar, Croatia; 6 Department for Cardiovascular Disease, Osijek University Hospital, J. Huttlera 4, HR-31000 Osijek, Croatia; 7 Department of Animal Production and Biotechnology, Faculty of Agrobiotechnical Sciences, Josip Juraj Strossmayer University of Osijek, Vladimira Preloga 1, HR-31000 Osijek, Croatia; 8 Department of Clinical Laboratory Diagnostics, Osijek University Hospital, J. Huttlera 4, HR-31000 Osijek, Croatia; seric.vatroslav@kbo.hr

**Keywords:** n-3 polyunsaturated fatty acids, omega-3 polyunsaturated fatty acids, endothelium, microvascular, inflammation, laser Doppler flowmetry, cytokines

## Abstract

The effects of consumption of n-3 polyunsaturated fatty acids (n-3 PUFAs) enriched hen eggs on endothelium-dependent and endothelium-independent vasodilation in microcirculation, and on endothelial activation and inflammation were determined in young healthy individuals. Control group (*N* = 21) ate three regular hen eggs/daily (249 mg n-3 PUFAs/day), and n-3 PUFAs group (*N* = 19) ate three n-3 PUFAs enriched hen eggs/daily (1053 g n-3 PUFAs/day) for 3 weeks. Skin microvascular blood flow in response to iontophoresis of acetylcholine (AChID; endothelium-dependent) and sodium nitroprusside (SNPID; endothelium-independent) was assessed by laser Doppler flowmetry. Blood pressure (BP), body composition, body fluid status, serum lipid and free fatty acids profile, and inflammatory and endothelial activation markers were measured before and after respective dietary protocol. Results: Serum n-3 PUFAs concentration significantly increased, AChID significantly improved, and SNPID remained unchanged in n-3 PUFAs group, while none was changed in Control group. Interferon-γ (pro-inflammatory) significantly decreased and interleukin-10 (anti-inflammatory) significantly increased in n-3 PUFAs. BP, fat free mass, and total body water significantly decreased, while fat mass, interleukin-17A (pro-inflammatory), interleukin-10 and vascular endothelial growth factor A significantly increased in the Control group. Other measured parameters remained unchanged in both groups. Favorable anti-inflammatory properties of n-3 PUFAs consumption potentially contribute to the improvement of microvascular endothelium-dependent vasodilation in healthy individuals.

## 1. Introduction

Endothelial dysfunction, which is characterized by a loss of vasodilator and anti-inflammatory properties of endothelium is considered as one of the earliest events in the pathological development of cardiovascular (CV) diseases [1]. n-3 polyunsaturated fatty acids (n-3 PUFAs) have the potential to prevent development and progression of various (CV) diseases, including atherosclerosis and atherosclerosis related diseases, in part, by improving vascular (endothelial) function as demonstrated in numerous epidemiological and clinical trials [2,3,4,5,6,7]. Some of these vasculo-protective properties include decreased blood pressure (BP) [8,9], decreased formation of atherosclerotic lesions [10], increased anti-inflammatory properties [11], improved endothelium-dependent vasodilation of conduit arteries, and increased antioxidant capacity [12].

Three different n-3 PUFAs have these protective effects: eicosapentaenoic acid (EPA) and docosahexaenoic acid (DHA) and alpha-linolenic acid (ALA). Common sources of n-3 PUFAs in daily diet are fatty fish (mackerel, salmon, sardines, trout etc.) and n-3 PUFAs rich vegetables, nuts and seeds (seaweed and algae, chia seeds, walnuts, kidney beans, etc.). Nevertheless, it is important to note that an important source of n-3 PUFAs are various n-3 PUFAs rich pharmacological supplements containing fish-oil, cod liver oil, krill oil, algae oil, or ALA supplements. A lot of research effort is ongoing, aiming to increase the content of n-3 PUFAs in different food stuff in the form of functional food. Food can be considered functional if in addition to appropriate nutritional effects, it also has beneficial effects on one or more target functions of the body [13]. Most commonly n-3 PUFAs functional foods available on the market include functional poultry products as hen eggs, fortified pasta, soy milk, oatmeal, cereal, margarine, etc. One of advantages of providing n-3 PUFAs in enriched hen eggs is that they are non-processed food, and they do not have “fishy” taste, which many consumers do not prefer [14].

Several studies demonstrated that n-3 PUFAs enriched functional foods (e.g., hen eggs) may reduce CV risk in CV patients [14,15,16]. On the other hand, although n-3 PUFAs functional products are widely available on the market, there is still insufficient data on their possible effects on CV health, and in particular vascular and endothelial function in healthy individuals. This would be very important to establish, given that millions of people around the world use n-3 PUFAs supplements every day (e.g., about 18.8 million adults in the United States take n-3 PUFAs fish oil supplements) [17]. For example, consumption of five n-3 PUFAs enriched hen eggs (241 mg n-3 PUFAs/egg) per week for 3 weeks significantly decreased serum triglycerides level (16–18% decrease) in 25 healthy individuals [18]. We have recently shown that consumption of three n-3 PUFAs enriched hen eggs per day (777 mg n-3 PUFAs/day) for 3 weeks enhanced skin microvascular reactivity to vascular occlusion and decreased arterial BP, blood triglycerides, and high-sensitivity C-reactive protein (hsCRP) level in young healthy population [19], suggesting that n-3 PUFAs may have anti-inflammatory properties, beneficial for vascular function.

**Figure 1:**
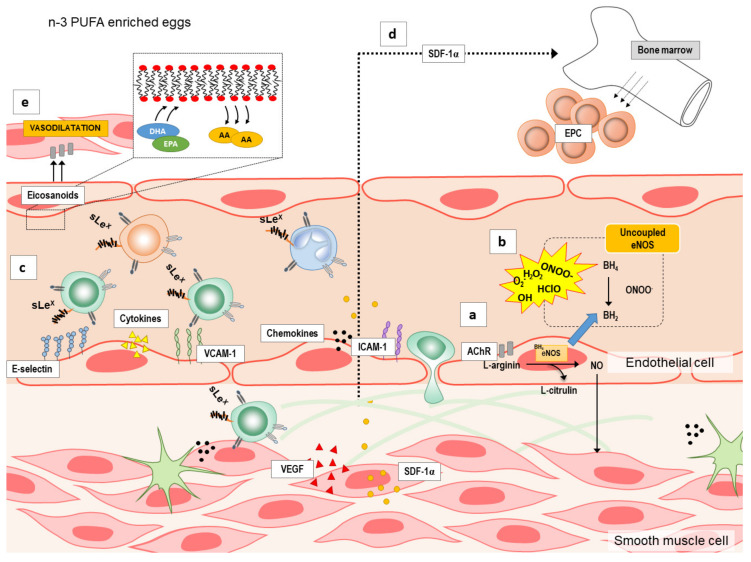
The summary of the potential mechanisms by which n-3 polyunsaturated fatty acids (n-3 PUFAs) supplementation may modulate endothelial function. Several possible targets by which n-3 PUFAs supplementation modulate endothelial function are: (**a**) increasing production of endothelium nitric oxide (NO), (**b**) reducing formation or increasing elimination of reactive oxygen species (ROS), (**c**) decreasing vascular or systemic inflammation (e.g., endothelial activation and endothelium–leukocyte interaction), (**d**) inducing angiogenesis/neovascularization and mobilization of bone-marrow derived endothelial progenitor cells (EPCs), and (**e**) increasing the expression and/or activity of other endothelium-derived vasodilators (e.g., eicosanoids). A—arachidonic acid; AChIR—acetylcholine induced relaxation; BH4—tetrahydrobiopterin; BH2—7,8-dihydrobiopterin; DHA—docosahexaenoic acid; eNOS—endothelial nitric oxide synthase; EPA—eicosapentaenoic acid; ICAM—1-intercellular adhesion molecule 1; n-3 PUFAs—n-3 polyunsaturated fatty acids; SDF-1α—stromal cell-derived factor 1; sLEx—Sialyl Lewisx; VCAM-1—vascular cell adhesion molecule 1; VEGF—vascular endothelial growth factor.

Although the mechanisms by which n-3 PUFAs supplementation may modulate endothelial function are under investigated, there are several possible targets (summarized in Figure 1): by increasing endothelium nitric oxide (NO) production (Figure 1a) by reducing reactive oxygen species (ROS) formation or increasing their elimination [20] (Figure 1b), and by decreasing vascular or systemic inflammation (Figure 1c) or inducing angiogenesis/neovascularization and mobilization of bone-marrow derived endothelial progenitor cells (EPCs) [21] (Figure 1d). n-3 PUFAs may increase NO either by activation of endothelial nitric oxide synthase (eNOS) or inducible nitric oxide synthase (iNOS) [22,23,24]. Moreover, in addition to NO, n-3 PUFAs can also increase the expression and/or activity of other endothelium-derived vasodilators [25] (Figure 1e). Lastly, endothelial dysfunction is also characterized by endothelial activation and inflammation, which may be attenuated by n-3 PUFAs anti-inflammatory potential [26,27,28,29] (Figure 1c), as suggested by studies in cell culture and animal studies. However, these potential mechanisms underlying the improvement of endothelial dysfunction by n-3 PUFAs supplementation in a form of functional food have not been fully investigated especially in healthy individuals.

In an effort to elucidate at least a part of the mechanisms by which n-3 PUFAs in functional food affects vascular function in healthy individuals, this study aimed: (a) to determine the effect of consumption of n-3 PUFAs enriched hen eggs on endothelium-dependent and endothelium-independent vasodilation of forearm skin microcirculation and (b) to examine its effect on various markers of endothelial activation and inflammation in young healthy individuals. In the EU, the average egg consumption is 217 eggs/citizen yearly, while in Croatia, 182.6 eggs/person have been eaten yearly [30]. Thus, we aimed to determine if consumption of significantly higher amount of eggs daily 3 eggs per day (including regular eggs) in a longer period presents itself as a CV risk.

## 2. Results

Participants’ initial anthropometric, hemodynamic, and biochemical characteristics are presented in Table 1. All participants were lean and normotensive, had normal full blood count, renal function, serum electrolytes, fasting blood glucose, hsCRP, and fasting lipid level. There was no significant difference in all measured parameters (e.g., age, BMI, BP, HR, and biochemical parameters) at the moment of entering the study protocol between participants who comprised Control and n-3 PUFAs group. All participants completed 3-weeks dietary protocol: the n-3 PUFAs group consumed n-3 PUFAs enriched hen eggs (total of 1053 n-PUFAs/day) and the Control group consumed regular hen eggs (total 249 n-PUFAs/day), both groups consumed 3 eggs per day.

### 2.1. Content of Fatty Acids in Chicken Feed Mixture and Edible Part of Eggs

Fatty acids profiles of chicken feed mixture (g/100 g of total fatty acids) and edible part of eggs (mg/100 g edible part) used in the present study are described in Table 2. Each n-3 PUFAs egg (average weight 60 g) contained on average 351 mg of n-3 PUFAs (ALA 230.5 mg/egg, EPA 15.1 mg/egg, DHA 105.5 mg/egg). Each control egg (average weight 60 g), produced on the same farm, contained on average 83 mg of n-3 PUFAs (ALA 36 mg/egg, EPA 0 mg/egg, DHA 47 mg/egg).

### 2.2. Anthropometric, Hemodynamic, and Biochemical Parameters

BMI, BP, HR, hsCRP, and serum lipid profile values in Control and n-3 PUFAs group before and after respective protocol are shown in Table 3. There was no significant difference in BMI after consumption of three n-3 PUFAs or regular hen eggs compared to baseline (initial) measurements within the n-3 PUFAs or Control group.

SBP and MAP significantly decreased, while DBP and HR values were similar after consumption of regular hen eggs compared to initial measurement in Control group. There was no significant change in SBP, DBP, MAP, and HR values before and after dietary protocol in n-3 PUFAs group. The values of SBP, DBP, MAP, and HR did not significantly differ between Control and n-3 PUFAs group before or after corresponding dietary protocols.

The values of hsCRP were not significantly changed by consumption of regular or n-3 PUFAs enriched eggs compared to baseline measurements within the groups, or there was no difference in hsCRP between the groups. There were no significant differences in serum total cholesterol, triglycerides, LDL cholesterol, and HDL cholesterol concentrations after regular or n-3 PUFAs enriched eggs consumption compared to baseline conditions within the groups. However, serum cholesterol and HDL cholesterol concentrations were slightly and significantly higher in Control group than in n-3 PUFAs group after completion of respective diet protocol (but still within the normal reference level).

### 2.3. Body Composition and Body Fluid Status

Body composition and body fluid status responses to dietary protocols are presented in Table 4. Fat% significantly increased, and Fat Free Mass% (FFM%) and Total Body Water% (TBW%) significantly decreased after consumption of three regular hen eggs during three weeks compared to baseline measurement in the Control group. There was no significant difference in FFM%, Fat%, TBW%, Extracellular Water% (ECW%), Intracellular Water% (ICW%), Plasma Fluid (PF), Interstitial Fluid (IF), or Body Density before and after dietary protocol within the n-3 PUFAs group. There was no difference in FFM%, Fat%, TBW%, ECW%, ICW%, PF, IF, or Body Density between the groups neither before nor after respective study protocols.

### 2.4. Serum Fatty Acids Profile

Serum fatty acids profile (a total of 37 fatty acids) in respect to dietary protocols is presented in Table 5. Serum concentration of ALA significantly increased, while EPA and DHA tended to increase following consumption of n-3 PUFAs enriched eggs compared to baseline measurement within the n-3 PUFAs group. Serum concentration of other measured fatty acids were similar before and after dietary protocol with n-3 PUFAs enriched eggs. As a result of n-3 PUFAs enriched hen eggs consumption, serum n-6/n-3 ratio decreased for approximately 30% in n-3 PUFAs group. Serum fatty acids profile was not significantly changed, and n-6/n-3 ratio decreased for approximately 16% following dietary protocol with regular eggs compared to baseline measurement in Control group. At baseline measurement prior entering the study, serum concentration of C16:0 palmitic acid, C18:0 stearic acid, C18:1[cis-9] oleic acid, C20:3[cis-8,11,14] dihomo-gamma-linolenic acid, and C20:4[cis-5,8,11,14] arachidonic acid was significantly higher in the Control compared to the n-3 PUFAs group. Serum DHA concentration was significantly increased, and C20:4[cis-5,8,11,14] arachidonic acid was significantly decreased in the n-3 PUFAs group compared to the Controls after completion of respective dietary protocol.

### 2.5. Forearm Skin Microvascular Endothelium-Dependent and Endothelium-Independent Vasodilation

**Figure 2:**
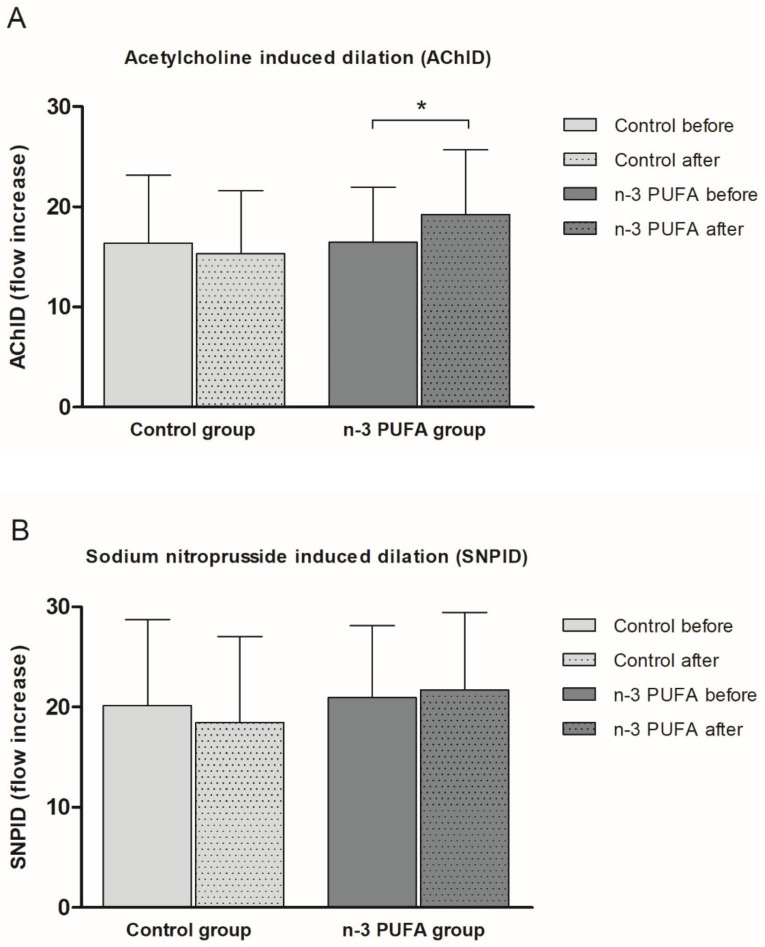
The effect of the three-week regular (Control group) and n-3 PUFAs (n-3 PUFAs group) enriched hen eggs consumption on skin microvascular endothelium-dependent and endothelium-independent vasodilation in young healthy individuals. (**A**) Acetylcholine-induced dilation (AChID), and (**B**) Sodium nitroprusside-induced dilation (SNPID). AChID and SNPID are expressed as flow increase following ACh or SNP administration compared to baseline flow. Data are presented as arithmetic mean ± standard deviation (SD). n-3 PUFA- n-3 polyunsaturated fatty acids. Control *N* = 21, n-3 PUFAs *N* = 19. * *p* = 0.014 before vs. after within the n-3 PUFA group (paired *t*-test).

Consumption of n-3 PUFAs enriched hen eggs significantly improved ACh-induced dilation (AChID) (an increase of 14.4%) (Figure 2A) of forearm skin microcirculation compared to baseline measurement within the n-3 PUFAs group. Contrarily, consumption of regular hen eggs did not induce any significant change in AChID of forearm skin microcirculation compared to baseline in Control group (Figure 2A). There was no significant difference in AChID at baseline or following respective hen eggs consumption between the groups (Figure 2A). SNP-induced dilation (SNPID) was similar between baseline and regular or n-3 PUFAs eggs consumption within the groups (Figure 2B), and similar between Control and n-3 PUFAs groups (Figure 2B).

### 2.6. Serum Pro- and Anti-Inflammatory Cytokines, Chemokines, Growth Factors, and Soluble Cell Adhesion Molecules Protein Concentration

Serum pro- (INFγ, TNF-α, IL-17A, IL-6, IL-23, and IL-9) and anti-inflammatory (and immunomodulatory) cytokines (IL-21, IL-22, and IL-10), chemokines (SDF-1α), LAP, vascular growth factors (VEGF-A, VEGF-D) and soluble cell adhesion molecules (sICAM-1, sVCAM-1), protein concentration values in the Control and n-3 PUFAs group before and after respective protocol are described in Table 6. INFγ protein concentration significantly decreased, and IL-10 serum concentration significantly increased following consumption of n-3 PUFAs enriched eggs compared to baseline measurement within the n-3 PUFAs group. Serum protein concentration of other measured cytokines, chemokines, growth factors, or soluble cell adhesion molecules (TNF-α, IL-17A, IL-6, IL-21, IL-22, IL-23, IL-9, SDF-1α, LAP, VEGF-A, VEGF-D, sICAM-1, and s-VCAM-1) were similar before and after dietary protocol with n-3 PUFAs enriched eggs. In Controls, IL-17A, IL-10, and VEGF-A significantly increased following consumption of regular hen eggs compared to the baseline measurement. Serum protein concentration of other measured cytokines, chemokines, growth factors or soluble cell adhesion molecules (INFγ, TNF-α, IL-6, IL-21, IL-22, IL-23, IL-9, SDF-1α, LAP, VEGF-D, sICAM-1, and s-VCAM-1) were similar before and after dietary protocol with regular eggs.

Only serum protein concentration of IL-10 was significantly increased in n-3 PUFAs group compared to the Controls after completion of respective dietary protocol. There was no other significant difference in serum protein values of measured cytokines, chemokines, growth factors or soluble cell adhesion molecules between the groups, before and after the diet protocols as shown in Table 6.

## 3. Discussion

This is the first randomized, double-blind, placebo-controlled interventional study which investigated the effect of n-3 PUFAs enriched hen eggs consumption on endothelial microvascular function, and their anti-inflammatory potential in young healthy individuals. First very important finding of this study is increased serum concentration of n-3 PUFAs in participants who consumed n-3 PUFAs enriched eggs, which demonstrates successful dietary protocol.

In particular, the novel finding of this study is that consumption of n-3 PUFAs enriched hen eggs improved endothelium-dependent vasodilation of skin microcirculation independently of BP or body composition and fluid status changes in healthy individuals. Furthermore, observed enhancement in endothelial function was accompanied by a significant increase in IL-10 (anti-inflammatory cytokine), and decrease in INFγ (pro-inflammatory cytokine) serum protein concentration. On the other hand, consumption of regular hen eggs did not significantly affect microvascular endothelial function, but significantly increased IL-17A (pro-inflammatory cytokine) and VEGF-A (vascular endothelial growth factor), as well as IL-10 serum protein concentration. Thus, our study suggests that changes in balance between pro-and anti-inflammatory cytokines could be a potential moderator of enhanced endothelial function following increased consumption of n-3 PUFAs in the form of functional food in healthy population. Furthermore, consumption of hen eggs per se, either regular or n-3 PUFAs, even three pieces a day for three weeks, did not have any harmful effects on metabolic (e.g., serum lipid profile; liver function not assessed), inflammatory or functional vascular parameters (LDF measurement of forearm skin microvascular reactivity) in participants of the study and did not increase CV risk in healthy subjects.

### 3.1. n-3 PUFAs Enriched Hen Eggs and Serum Lipid Profile

n-3 PUFAs supplementation (EPA and DHA) can reduce serum lipids, most notably triglycerides in hyperlipidemic individuals [31,32]. In the present study, consumption of n-3 PUFAs enriched hen eggs did not induce significant change in serum lipid profile compared to baseline measurement; triglycerides level decreased by approximately 8% (*p* > 0.05). At the end of the protocol, total cholesterol and HDL cholesterol level were slightly higher in Control group than in n-3 PUFAs group. However, this difference is statistically rather than clinically relevant, since both total and HDL cholesterol levels were within the normal reference range. Thus, we conclude that consumption of 3 eggs per day does not pose risk for unfavorable changes in serum lipid profile for healthy individuals. Our results are consistent with the results of a meta-analysis by Leslie et al. who reported that only very high doses of EPA and/or DHA-enriched food sources (≥4 g/day) have the ability to reduce serum triglycerides (by 9–26%) in normolipidemic to borderline hyperlipidemic and otherwise healthy individuals [33]. Several studies demonstrated that consumption of n-3 PUFAs enriched hen eggs has the potential to decrease serum triglycerides (but not total, HDL and LDL cholesterol levels) in healthy individuals [18,19,34,35], but the lack of consistent study design for elevating n-3 PUFAs through dietary modifications, particularly functional food, continues to be a limitation in the field. Interestingly, it has been suggested that higher doses of EPA and DHA may be required to decrease serum triglycerides then are needed to improve endothelial dysfunction [2], and furthermore, endothelial function may be improved in the absence of a decrease in triglycerides [2], which is in accordance with the present results.

### 3.2. n-3 PUFA Enriched Hen Eggs, Blood Pressure Level, Body Composition, and Body Fluid Status

n-3 PUFAs supplementation may induce clinically relevant BP decrease in patients with untreated hypertension [36], in patients with essential hypertension [37,38], and in mildly hypercholesterolemic but normotensive individuals [39]. However, such significant effect of n-3 PUFAs in lowering BP level was not observed in normotensive individuals [40,41], and was not observed in the present study. For example, supplementation of n-3 PUFAs in the form of four fish servings per week (∼800 mg/serving EPA+DHA for 8 weeks) or fish oil supplementation (2 g/day EPA+DHA for 12 weeks; 1.7 g/day EPA+DHA for 4 weeks) in normotensive individuals [42,43,44] did not significantly change BP. Still, our earlier study demonstrated that consumption of n-3 PUFAs enriched hen eggs (777 mg/day) for three weeks significantly reduce BP in healthy normotensive individuals, similar to study of Oh et al. [34]. Surprisingly, BP level significantly decreased in healthy individuals who consumed three regular hen eggs for three weeks. Thus, decrease in BP could not be attributed to n-3 PUFAs in present study. 

The influence of n-3 PUFAs intake on body composition is unclear. One systematic review of clinical trials did not find any important effect of dietary n-3 PUFAs intake on body weight in mostly overweight and obese adults [45]. Another meta-analysis of only randomized clinical trials that explicitly examined body composition-related measures and used only n-3 PUFAs of fish provenience reported minimal non-significant change in body weight (590 g) and other body composition-related outcomes (such as BMI, body fat percentage and waist circumference) between intervention and control groups [46]. Even consumption of seven n-3 PUFAs enriched hen eggs per week for 24 weeks did not cause any significant change in body weight compared to baseline in healthy volunteers [47]. Consistently, in the present study we did not observe any significant change in measured body composition parameters and body fluid status following consumption of n-3 PUFAs enriched hen eggs in healthy lean individuals. Interestingly, body fat mass significantly increased, and fat free mass as well as total body water significantly decreased following consumption of regular hen eggs in the Controls, an observation for which the mechanism is still not clear.

Since in the present study n-3 PUFAs consumption was not accompanied by changes in serum lipid profile, BP level, body composition and body fluid, it seems plausible that improved microvascular endothelial function due to n-3 PUFAs intake is a consequence of the unique effect of increased serum n-3 PUFAs concentration, decreased n-6/n-3 PUFAs ratio, and favorable anti-inflammatory milieu rather than systemic hemodynamic and/or obesity-related changes in healthy individuals, which will be further discussed.

### 3.3. n-3 PUFA Enriched Hen Eggs and Microvascular Endothelial Function

n-3 PUFAs have the potential to modify vascular endothelial function by being incorporated into endothelial cell membrane phospholipids (at the expense of n-6 PUFAs, such as arachidonic acid), resulting in modulation of different factors which determine endothelial function itself (e.g., synthesis of vasoactive mediators derived from endothelium, oxidative stress level, endothelial activation and inflammation etc.) [48]. Present study demonstrated significant decrease in serum arachidonic acid at the expense of increased n-3 PUFAs, which may be a source for anti-inflammatory and vasoprotective eicosanoids, contributing to enhanced microvascular response, as observed.

A number of multiple epidemiological, experimental and clinical studies in patients with increased CV risks or already existing CV diseases, such as hypertriglyceridemia, peripheral artery disease, diabetes mellitus type 2, healthy smokers etc. reported that n-3 PUFAs may reduce the risk of CV diseases, at least in part, by improving vascular function [2]. Detailed overview of these studies in CV patients was recently presented in two reviews by Zehr and Walker [2], and Du et al. [49], respectively. Importantly, referenced studies utilized pharmacological supplementation of n-3 PUFAs in form of capsules, but not functional food.

The present study demonstrated, for the first time, that peripheral microvascular endothelial-dependent vasodilation, but not endothelial-independent is improved in response to n-3 PUFAs enriched hen eggs consumption in young healthy individuals. Our results are in agreement with improved endothelium-dependent vasodilation following iontophoretic applications of ACh and SNP in healthy individuals who took fish oil supplementation (EPA + DHA) for 8 months [50]. In contrast, a randomized cross-over designed study of three diets, each lasting for 4 weeks reported that postprandial forearm skin post-occlusive reactive hyperemia was significantly increased only after monounsaturated fatty acids-rich Mediterranean diet, but not low-fat diet enriched in ALA in 20 healthy men [51]. On the other hand, previously we reported that young healthy subjects who consumed 777 mg of n-3 PUFAs/day in enriched eggs for 3 weeks had improved skin microvascular reactivity in response to PORH [19]. All together, these results suggest that a threshold dose of n-3 PUFAs in food/serum needs to be achieved to observe effects in microcirculatory vasodilator responses.

### 3.4. n-3 PUFAs Enriched Hen Eggs and Pro- and Anti-inflammatory Cytokines, Chemokines, Growth Factors, and Soluble Cell Adhesion Molecules

Still, mechanisms mediating endothelium-dependent vasodilation in both micro- and macrovasculature, both in patients and healthy individuals are not known. It has been suggested that n-3 PUFAs may improve endothelial function by increasing the bioavailability of main endothelial vasodilator NO (increasing NO production and/or decreasing oxidative stress level) (Figure 1a), and/or by changing the expression/activity of other endothelial-derived vasoactive mediators, in particular metabolites of arachidonic acid (e.g., COX metabolites PGI2, PGH2, TXA2; CYP450 metabolites epoxyeicosatrienoic acids, EETs; and 20-hydroxyeicosatetraenoic acid, 20-HETE) (Figure 1e) [12,22,23,25,52,53].

Additionally, inflammatory stimuli, such as cytokines and chemokines can activate endothelium, as an early event in the initiation of adhesion of monocytes and other leukocytes to the endothelium, preceding their infiltration and extravasation to the site of injury [54]. Activated endothelium interacts with blood leukocytes, which is characterized by increased expression of cell adhesion molecules (e.g., ICAM-1, and VCAM-1), and increased secretion of chemokines (e.g., LAP, SDF-1α) and pro-inflammatory cytokines (e.g., IL-6 and TNF-α), and with the progression of inflammation with a release of variety of vascular growth factors (VEGF-A and VEGF-D), which under certain circumstances may act as angiogenic mediators that promote atherogenic plaque progression and instability (Figure 1c,d). On the other hand, VEGF is a potent growth factor for endothelial cells and inducer of angiogenesis, important for endothelial integrity and thus for vascular function [55]. A previous study by Wu and colleagues showed that fish-oil supplementation enhanced mobilization of bone-marrow derived EPCs independently of eNOS genotype in patients with moderate risk for CV diseases [21]. In the present study, SDF-1 serum levels were unaffected by diets, suggesting there were no alterations in EPCs numbers in the blood, possibly due to non-existent or low risk for CV diseases in our cohort.

There is a good evidence that n-3 PUFAs can decrease (or prevent) endothelium–leukocyte interactions by decreasing the expression of cell adhesion molecules (ICAM, VCAM, and/or E-selectin) [54,56]. The few animal feeding studies in mice and rats, all reported reduced VCAM-1 expression after feeding with EPA and/or DHA. In healthy individuals, n-3 PUFAs may reduce levels of soluble adhesion molecules (e.g., reduced sICAM-1 after supplementation of 2–6.6 g of EPA + DHA daily for 8–12 weeks; reduced sVCAM-1 after supplementation of 1 g EPA + DHA for 12 weeks) [57,58,59]. However, there are also reports on no change (e.g., no change in sICAM-1 and sVCAM-1 after 1.35, 2.7, or 4.05 g EPA daily for 12 weeks in healthy young and older men) [60], or even increase in sCAMs following n-3 PUFAs intake (e.g., increased sVCAM-1 after 1.3 g DHA and 700 mg EPA daily for 8 weeks in healthy men and women [61]; increase in sICAM-1 after 1.37 g EPA and 240 mg DHA daily for 8 weeks in healthy individuals) [62]. The present study demonstrated that consumption of n-3 PUFAs enriched hen eggs (but also of regular eggs) did not induce significant change in sICAM-1 and sVCAM-1 levels in healthy individuals. Thus, we may exclude endothelial activation as a mechanism of action.

Additionally, n-3 PUFAs may be able to influence the levels of circulatory pro- and anti-inflammatory molecules (cytokines, chemokines, and growth factors) directly. n-3 PUFAs intake is associated with reduced concentrations of acute phase protein reactants (CRP), pro-inflammatory eicosanoids, cytokines, chemokines, and other inflammation biomarkers [56]. Moreover, in addition to inhibiting pro-inflammatory mediators, some animal studies also report that n-3 PUFAs may reciprocally increase the concentration of the anti-inflammatory cytokine IL-10 [63,64]. While the results on the effect of n-3 PUFAs on inflammatory molecules in population with increased CV risk remain inconclusive, majority of studies in healthy individuals demonstrated that n-3 PUFAs did not significantly affect the serum concentration of any of the cytokines (e.g., IL-1α, IL-1β, IL-2, IL-4, IL-5, IL-6, IL-8, IL-10, IL-12p70, IL-13, TNF-α, and INFγ) and chemokines (e.g., CCL2, CCL3, CCL5, and CCl11) measured in these studies [56]. On the other hand, fish oil supplements to healthy individuals decreased the production of TNF-α, IL-1β, and IL-6 by endotoxin-stimulated monocytes or mononuclear cells [65]. Potential reason may be an insufficient dose of provided n-3 PUFAs (<2 g EPA + DHA), together with basally low levels of cytokines and chemokines in serum of healthy subjects. The latter is supported by the fact that anti-inflammatory effects of n-3 PUFAs have been repeatedly confirmed in studies employing individuals suffering from autoimmune or inflammatory disorders [66]. To our knowledge, the present study is the first study to report that functional food rich in n-3 PUFAs decreased INFγ and increased IL-10 serum concentration in healthy individuals. Furthermore, consumption of regular hen eggs for three weeks increased IL-10 (less than observed in n-3 PUFA group), but also increased IL-17A and VEGF-A serum concentration in healthy individuals. These results may indicate that unlike the uptake of n-6 PUFAs which leads to an increase in the concentration of pro-inflammatory cytokines (such as IL-17A) and angiogenic growth factors (VEGF-A), uptake of n-3 PUFAs may contribute to maintaining a favorable anti-inflammatory milieu in healthy individuals.

## 4. Materials and Methods

### 4.1. Study Population

Forty young healthy individuals (20 women and 20 men) participated in this study. Eligibility criteria included age range between 18 and 30 years, normal body mass index (BMI), arterial BP values, and serum lipid ranges. Exclusion criteria were a history of smoking, hypertension, coronary artery disease, diabetes, hyperlipidemia, renal impairment, cerebrovascular and peripheral artery disease, and taking any drugs or substances that could affect the endothelium. Also, none of the participants had been taking n-3 PUFAs enriched functional food nor n-3 PUFAs supplementation in the form of capsules prior to enrollment in the present study. Written informed consent was obtained from each subject. The study protocol and procedures conformed with the standards set by the latest revision of the Declaration of Helsinki and were approved by the Ethical Committee of the Faculty of Medicine, University of Osijek (Cl: 602-04/14-08/06; No: 2158-610714-114).

### 4.2. Production of n-3 PUFAs Enriched Hen Eggs and Assessment of Fatty Acids Profile of Chicken Feed Mixtures and Edible Part of Eggs

n-3 PUFAs enriched hen eggs were produced according to the protocol of research group from Faculty of Agrobiotechnical Sciences Osijek, Josip Juraj Strossmayer University of Osijek, in which soybean oil (5%) in feed mixtures fed to laying hens were replaced with mixture of fish (1.5%) and linseed (3.5%) oil.

Preparation and analysis of samples for determination of fatty acids was performed according to the method of Csapo et al. [67]. Gas chromatography was performed on a Bruker 430-GC apparatus (Billerica, MA, USA), equipped with a FAMEWAX (RESTE; Bellefonte, PA, USA) type capillary column (30 m × 0.32 mm internal diameter, 0.25 µm film) and flame ionization detector. Characteristic operating conditions were: injector temperature: 220 °C, detector temperature: 230 °C, helium flow: 25 mL/min. The oven temperature was graded: from 50 to 225 °C: 6.0 °C/min, 21 min at 225 °C. To identify individual fatty acids in the chromatogram, a fatty acid standard mixture Supelco 37 Component FAME Mix (Supelco Inc.; Bellefonte, PA, USA) was used. Portions of saturated fatty acid (SFA) and monounsaturated fatty acid (MUFA), as well as n-6 PUFAs and n-3 PUFAs were shown as a g/100 g of total fatty acids in feed-mixtures, and in mg/100 g in eggs (edible part).

### 4.3. Study Protocol

This was a randomized, double-blind, placebo-controlled interventional study (registration on ClinicalTrials, https://clinicaltrials.gov/, Identifier: NCT02720250). Study protocol lasted for three weeks (21 day). During those three weeks’ subjects were instructed to eat three hen eggs per day (total of 63 eggs). According to the type of eggs they ate, subjects were divided in two study groups: control (Control; 21 subjects) and experimental (n-3 PUFAs; 19 subjects). n-3 PUFAs group consumed n-3 PUFAs enriched hen eggs (three per day; around 1053 mg of n-3 PUFA per day), while Control group consumed regular hen eggs produced on the same farm (three per day; around 249 mg of n3 PUFA per day). Neither the researcher nor the subjects knew which group the subjects belong to until the end of the three weeks’ dietary protocol (eggs were labeled #1 or #2 before distributed to the Laboratory). n-3 PUFAs enriched eggs and regular hen eggs were the same size (M commercial size). Subjects were instructed to boil the eggs for about 10 min before consumption. Also, all subjects were instructed to take only the eggs given to them for the purposes of the study (total of 63 eggs) and not to take the other food rich in n-3 PUFAs, n-3 PUFAs enriched functional food or any other form of n-3 PUFAs supplementation during study protocol. The study was performed in the Laboratory for Clinical and Sport Physiology, Department of Physiology and Immunology at Faculty of Medicine, University of Osijek. Two study visits and all the measurements described below were done on the first day and the day immediately after the end of the protocol. All testing occurred in the morning after an overnight fasting. Subjects were instructed not to undertake any strenuous activity during the 24 h preceding the visit and to avoid caffeine intake in the morning before the study visit.

### 4.4. Anthropometric and Arterial Blood Pressure Measurements 

Subjects height (m) and weight (kg) were measured to calculate body mass index (BMI). BP and heart rate (HR) were measured at the beginning of each visit after a 15 min rest in a seated position using an automated oscillometric sphygmomanometer (OMRON M3, OMRON Healthcare Inc.; Osaka, Japan). The final values of BP and HR were the mean of three repeated measurements.

### 4.5. Body Composition and Body Fluid Status Measurements

Body composition and body fluid status were measured using a four-terminal portable impedance analyzer (Maltron Bioscan 920-II, Maltron International Ltd.; Rayleigh, Essex, UK). Empirically derived formulas (the original manufacturer’s software) were used to calculate the estimated Fat Free Mass% (FFM%), Fat% (Fat Mass%), Total Body Water% (TBW%), Extracellular Water% (ECW%), Intracellular Water% (ICW%), Plasma Fluid (PF), Interstitial Fluid (IF), and Body Density (kg/L).

### 4.6. Venous Blood Samples Analysis

A venous blood sample was taken after 15 min resting in a seated position at each visit. Blood samples were analyzed for full blood count, plasma electrolytes (sodium, potassium), urea, creatinine, fasting lipid profile (total cholesterol, high-density lipoprotein (HDL) cholesterol, low-density lipoprotein (LDL) cholesterol, and triglycerides), fasting blood glucose and hsCRP using standard laboratory methods. Venous blood samples analyses were performed at the Department of Clinical Laboratory Diagnostics, University Hospital Osijek.

### 4.7. Analysis of Serum Fatty Acids Profile

Gas chromatography–tandem mass spectrometry (GC–MS/MS) technique was applied for analysis of serum fatty acids profile. 37 component fatty acid methyl esters (FAME MIX) stock solution were purchased as 30 mg/mL total concentration of fatty acids in methylene chloride from Supelco (Supelco Inc.; Bellefonte, PA, USA), and used for the preparation of standard solutions. The sample preparation procedure from Wang et al. [68] was modified and used for analysis. For serum fatty acids profile analysis, the GC-MS/MS system by Thermo Fisher GC Trace 1300 coupled with a TSQ 9000 Triple Quadrupole was used. Serum fatty acids profile analysis was performed at the BIOCentre’s Bioanalytical Laboratory, BIOCentre - incubation center for biosciences, Zagreb, Croatia.

### 4.8. Assessment of Microvascular Endothelium-Dependent and Endothelium-Independent Vasodilation

Laser Doppler flowmetry (LDF) (MoorVMS-LDF, Axminster, UK) was used to assess an endothelium-dependent and endothelium-independent vasodilation of forearm skin microcirculation by iontophoresis (noninvasive transdermal application of charged substances) of acetylcholine (ACh) and sodium nitroprusside (SNP), respectively. LDF measurements were performed at each study visit in a temperature-controlled room (mean ± SD temperature = 23.5 ± 0.5 °C). Data collection started 30 min after resting in a supine position to acclimatize. The laser Doppler probe was attached to the skin of the volar forearm, 13–15 cm from the wrist, at the same place at each study visit using doubled sided adhesive discs provided by the manufacturer. Substances for iontophoresis were placed in an iontophoretic drug-delivery electrode that was attached at the site of the LDF probe. After baseline recording for 5 min, either the positively charged vasodilator ACh (1%) was iontophorezed with anodal current applied by means of seven pulses of direct electric current of 0.1 mA for 30 s with 30 s between each dose, or negatively charged SNP (1%) was applied by means of three pulses of 0.1 mA of negative current for 30 s, followed by a four pulses of 0.2 mA for 30 s, with 90 s between each dose. The pulsed iontophoretic protocols are adapted to obtain a stable plateau of the maximal LDF response. Microcirculatory blood flow was expressed in arbitrary perfusion units and determined by software calculating the area under the curve (AUC) during baseline flow and during ACh or SNP administration. The result was expressed as a blood flow increase following ACh or SNP administration in relation to baseline flow (ACh or SNP blood flow increase) [69,70,71].

### 4.9. Measurement of Serum Pro- and Anti-Inflammatory Cytokines, Chemokines, Growth Factors, and Soluble Cell Adhesion Molecules Protein Concentration

Serum protein concentration of pro-inflammatory cytokines: interferon gamma (INFγ), tumor necrosis factor alpha (TNF-α), interleukin 17A (IL-17A), interleukin 6 (IL-6), interleukin 23 (IL-23) and interleukin 9 (IL-9); anti-inflammatory cytokines and immunomodulatory cytokines: interleukin 21 (IL-21), interleukin 22 (IL-22), interleukin 10 (IL-10); chemokines: stromal cell-derived factor 1 alpha (SDF-1α); latency associated peptide (LAP), vascular endothelial growth factor A (VEGF-A), vascular endothelial growth factor D (VEGF-D), soluble intercellular adhesion molecule 1 (sICAM-1) and soluble vascular cell adhesion molecule 1 (sVCAM-1) were measured with Invitrogen ProcartaPlex antibody-based, magnetic bead reagent kits and panels for multiplex protein quantitation using the Luminex 200 instrument platform. Measurements were performed in Laboratory for Immunology and Allergology Diagnostics Osijek University Hospital, Osijek, Croatia. Quantitation was done in ProcartaPlex Analyst free software and expressed as concentration in picograms per milliliter.

### 4.10. Statistical Analysis

All results are reported as the arithmetic mean ± standard deviation (SD). Differences between measurements done before and after respective diet protocol within groups were assessed using paired t-test, or the Wilcoxon rank-sum test when variables were not normally distributed. To assess differences between Control and n-3 PUFAs group, Student t-test for parametric and Mann–Whitney test for nonparametric distributions were used. The normality of data distribution was assessed by the Kolmogorov–Smirnov normality test. *p* < 0.05 was considered statistically significant. SigmaPlot, version 11.2 (Systat Software, Inc., Chicago, IL, USA) was used for statistical analysis.

## 5. Conclusions

In conclusion, this study provides functional evidence that n-3 PUFAs from functional food (n-3 PUFAs enriched hen eggs) can increase serum free n-3 PUFAs concentration and to improve microvascular endothelium-dependent vasodilation, while decreasing serum pro-inflammatory (e.g., INFγ), and increasing serum anti-inflammatory cytokines (IL-10) in young healthy subjects. Thus, it provides first potential mechanisms for enhanced microvascular function. 

Since consumption of regular hen eggs was associated with an increase in pro-inflammatory but also immunomodulatory cytokines and accompanied by unchanged endothelium-dependent dilation, these results suggest the anti-inflammatory potential of n-3 PUFAs enriched functional food in improving microvascular endothelial function of healthy individuals, but also that regular consumption of hen eggs does not carry a risk for increased microvascular dysfunctions and inflammation in healthy individuals.

## Figures and Tables

**Table 1:** Initial clinical characteristics of study population.

Parameter	Control	n-3 PUFAs
N (W/M)	21 (10/11)	19 (10/9)
Age (years)	24 ± 3	24 ± 2
BMI (kg/m2)	24.3 ± 3.1	23.3 ± 3.6
SBP (mmHg)	117 ± 10	115 ± 11
DBP (mmHg)	77 ± 8	77 ± 8
MAP (mmHg)	90 ± 7	90 ± 7
HR (beats per min)	77 ± 11	78 ± 13
erythrocytes (× 10E12/L)	4.8 ± 0.3	4.7 ± 0.4
hemoglobin (g/L)	140 ± 11	142 ± 13
hematocrit (%)	41.0 ± 2.8	41.5 ± 3.4
leukocytes (× 10E9/L)	6.2 ± 1.4	6.3 ± 1.4
thrombocytes (× 10E9/L)	256 ± 66	225 ± 36
urea (mmol/L)	5.3 ± 1.3	5.7 ± 1.3
creatinine (µmol/L)	79 ± 17	83 ± 18
sodium (mmol/L)	138 ± 2	138 ± 2
potassium (mmol/L)	4.1 ± 0.2	4.2 ± 0.2
glucose (mmol/L)	4.8 ± 0.6	4.6 ± 0.8
hsCRP (mg/L)	1.6 ± 2.2	1.3 ± 1.2
cholesterol (mmol/L)	5.2 ± 0.9	4.6 ± 0.9
triglycerides (mmol/L)	1.1 ± 0.5	1.3 ± 1.0
HDL cholesterol (mmol/L)	1.5 ± 0.4	1.3 ± 0.2
LDL cholesterol (mmol/L)	3.1 ± 0.7	2.7 ± 0.6

Data are presented as mean ± standard deviation (SD). n-3 PUFAs—n-3 polyunsaturated fatty acids; N—number of participants; W—women; M—men; BMI—body mass index; SBP—systolic blood pressure; DBP—diastolic blood pressure; MAP—mean arterial pressure; HR—heart rate; hsCRP—high-sensitivity C reactive protein; HDL—high-density lipoprotein; LDL—low-density lipoprotein.

**Table 2:** Fatty acids profile of hens feeding mixture and edible parts of eggs.

Fatty Acid	Feeding Mixture (*n* = 3)		Eggs (*n* = 10)	
	(g/100 g Total Fatty Acids)		(mg/100 g Egg ^1^)	
	Control	n-3 PUFAs	Control	n-3 PUFAs
∑SFA	16.6 ± 0.2	16.8 ± 0.2	2082.6 ± 83.1	2162.1 ± 52.6
∑MUFA	26.5 ± 0.1	25.9 ± 0.1	2669.6 ± 84.8	2917.5 ± 137.9 *
∑n-6 PUFA	51.9 ± 0.1 *	23.4 ± 0.2	1417.7 ± 119.3 *	1182.6 ± 111.9
LA	51.9 ± 0.1 *	23.2 ± 0.2	1274.4 ± 127.3	1106.6 ± 108.6
AA	N/F	0.17 ± 0.01	125.4 ± 6.1 *	67.1 ± 3.6
∑N-3 PUFA	5.05 ± 0.04	33.9 ± 0.3 *	138.2 ± 28.4	585.2 ± 77.1 *
ALA	5.05 ± 0.04	28.5 ± 0.2 *	59.9 ± 16.1	384.2 ± 64.0 *
EPA	N/F	1.87 ± 0.03	N/F	25.2 ± 3.2
DHA	N/F	3.54 ± 0.07	78.3 ± 13.8	175.8 ± 25.9 *
∑n-6/n-3 PUFA	10.3 *	0.69	10.3 *	2.02

Data are presented as mean ± standard deviation (SD). n-3 PUFA—n-3 polyunsaturated fatty acids; n—number of analysis; ∑SFA—saturated fatty acids (C14:0, C15:0, C16:0, C17:0, C18:0, C20:0, C21:0, C23:0); ∑MUFA—monounsaturated fatty acids (C14:1, C16:1, C18:1n9t, C18:1n9c, C20:1n9, C22:1n9); ∑n-6 PUFA—polyunsaturated fatty acids (C18:2n6c, C18:3n6, C20:3n6, C20:4n6, C22:2n6); LA—linoleic acid (C18:2n6c); AA—arachidonic acid (C20:4n6); ∑n-3 PUFA—polyunsaturated fatty acids (C18:3n3, C20:3n3, C20:5n3, C22:6n3); ALA—alpha linolenic acid (C18:3n3); EPA—eicosapentaenoic acid (C20:5n3); DHA—docosahexaenoic acid (C22:6n3); N/F—not found. ^1^ edible part; * *p* < 0.05 control vs. n-3 PUFAs.

**Table 3:** The effect of regular (Control group) and n-3 PUFAs enriched hen eggs (n-3 PUFAs group) consumption on anthropometric, hemodynamic, and biochemical parameters.

Parameter	Control		n-3 PUFAs	
	Before	After	Before	After
BMI (kg/m2)	24.3 ± 3.1	23.0 ± 6.1	23.3 ± 3.6	21.9 ± 6.8
SBP (mmHg)	117 ± 10 †	112 ± 11	115 ± 11	112 ± 11
DBP (mmHg)	77 ± 8	73 ± 8	77 ± 8	74 ± 8
MAP (mmHg)	90 ± 7 †	86 ± 8	90 ± 7	87 ± 8
HR (beats per min)	77 ± 11	73 ± 11	78 ± 13	76 ± 11
hsCRP (mg/L)	1.6 ± 2.2	1.8 ± 2.6	1.3 ± 1.2	1.8 ± 2.1
cholesterol (mmol/L)	5.2 ± 0.9	5.3 ± 1.0 *	4.6 ± 0.9	4.7 ± 0.7
triglycerides (mmol/L)	1.1 ± 0.5	1.1 ± 0.4	1.3 ± 1.0	1.2 ± 0.6
HDL cholesterol (mmol/L)	1.5 ± 0.4	1.5 ± 0.4 *	1.3 ± 0.2	1.3 ± 0.2
LDL cholesterol (mmol/L)	3.1 ± 0.7	3.1 ± 0.8	2.7 ± 0.6	2.8 ± 0.4

Data are presented as mean ± standard deviation (SD). n-3 PUFA—n-3 polyunsaturated fatty acids; BMI—body mass index; SBP—systolic blood pressure; DBP—diastolic blood pressure; MAP—mean arterial pressure; HR—heart rate; HDL–high-density lipoprotein; LDL—low-density lipoprotein. * *p* < 0.05 difference between Control and n-3 PUFA group; † *p* < 0.05 difference between before and after within the group.

**Table 4:** Body composition and body fluid status responses to regular (Control group) and n-3 PUFAs enriched hen eggs (n-3 PUFAs group) consumption.

Parameter	Control		n-3 PUFAs	
	Before	After	Before	After
Fat Free Mass (%)	79.1 ± 8.6 *	75.7 ± 8.4	77.7 ± 6.9	77.8 ± 6.7
Fat (%)	20.9 ± 8.6 *	24.3 ± 8.4	22.3 ± 6.9	22.2 ± 6.7
Total Body Water (%)	56.7 ± 5.4 *	54.2 ± 5.8	56.6 ± 6.2	55.5 ± 4.8
Extracellular Water (%)	43.0 ± 4.1	42.8 ± 4.4	41.4 ± 1.2	41.3 ± 1.4
Intracellular Water (%)	57.0 ± 4.1	57.2 ± 4.4	58.6 ± 1.2	58.7 ± 1.4
Plasma Fluid (L)	3.64 ± 0.68	3.56 ± 0.72	3.46 ± 0.96	3.34 ± 0.89
Interstitial Fluid (L)	12.73 ± 2.36	12.44 ± 2.51	12.11 ± 3.38	11.68 ± 3.12
Body Density (kg/L)	1.051 ± 0.020	1.044 ± 0.018	1.048 ± 0.015	1.048 ± 0.014

Data are presented as mean ± standard deviation (SD). n-3 PUFA- n-3 polyunsaturated fatty acids. * *p* < 0.05 difference between before and after within the group.

**Table 5:** The effect of regular (Control group) and n-3 PUFAs enriched hen eggs (n-3 PUFAs group) consumption on serum fatty acids profile.

Parameter	Control			n-3 PUFAs	
	Before	After		Before	After
SFA (μmol/L)					
C4:0 Butyric acid	N/F		N/F	N/F	N/F
C6:0 Caproic acid	N/F		N/F	N/F	N/F
C8:0 Caprylic acid	N/F		N/F	N/F	N/F
C10:0 Capric acid	N/F		N/F	N/F	N/F
C11:0 Undecylic acid	N/F		N/F	N/F	N/F
C12:0 Lauric acid	<LOQ		<LOQ	<LOQ	<LOQ
C13:0 Tridecylic acid	N/F		N/F	<LOQ	<LOQ
C14:0 Myristic acid	35.0 ± 14.5		25.5 ± 5.6	42.0 ± 12.3	32.7 ± 5.2
C15:0 Pentadecylic acid	12,70		<LOQ	<LOQ	10,90
C16:0 Palmitic Acid	825.4 ± 127.6 ^†^		767.1 ± 185.1	515.2 ± 170.5	552.7 ± 253.8
C17:0 Margaric acid	9,91		<LOQ	10,80	10.3 ± 0.8
C18:0 Stearic acid	224.9 ± 40.2 ^†^		213.8 ± 36.4	153.1 ± 61.0	161.1 ± 78.6
C20:0 Arachidic acid	<LOQ		<LOQ	<LOQ	<LOQ
C21:0 Heneicosanoic acid	N/F		N/F	N/F	N/F
C22:0 Behenic acid	<LOQ		<LOQ	<LOQ	<LOQ
C23:0 Tricosanoic acid	<LOQ		<LOQ	<LOQ	<LOQ
C24:0 Lignoceric acid	<LOQ		<LOQ	<LOQ	<LOQ
PUFA (μmol/L)					
n-5					
C14:1[cis-9] Myristoleic acid	<LOQ		<LOQ	13,7	<LOQ
C15:1[cis-10] Cis-10-Pentadecenoic acid	<LOQ		<LOQ	<LOQ	<LOQ
n-7					
C16:1[cis-9] Palmitoleic acid	61.9 ± 26.5		53.7 ± 22.2	47.9 ± 19.3	45.5 ± 19.1
C17:1[cis-10] cis-10-Heptadecenoic acid	8,90		<LOQ	5,50	<LOQ
n-9					
C18:1[trans-9] Elaidic acid	N/F		N/F	N/F	N/F
C18:1[cis-9] Oleic acid	562.0 ± 108.2 ^†^		507.1 ± 151.2	372.4 ± 152.7	383.6 ± 235.4
C20:1[cis-11] 11-Eicosenoic acid	<LOQ		<LOQ	<LOQ	<LOQ
C22:1[cis-13] Erucic acid	N/F		N/F	<LOQ	<LOQ
C24:1[cis-15] Nervonic acid	N/F		N/F	<LOQ	<LOQ
n-6					
C18:2[trans-9,12] Linoelaidic acid	N/F		N/F	N/F	N/F
C18:2[cis-9,12] Linoleic acid	1065.4 ± 136.8		961.0 ± 95.1	849.1 ± 291.6	889.3 ± 363.8
C18:3[cis-6,9,12] gamma-Linolenic acid	21.0 ± 5.2		16.9 ± 4.1	18.2 ± 5.0	16.4 ± 6.6
C21:2[cis-11,14] Eicosadienoic acid	7.1 ± 0.5		7,40	8,10	7,8
C20:3[cis-8,11,14] Dihomo-gamma-linolenic acid	55.4 ± 15.9 ^†^		45.5 ± 10.8	29.8 ± 8.3	32.1 ± 11.1
C20:4[cis-5,8,11,14] Arachidonic acid	355.2 ± 91.4 ^†^		363.8 ± 74.6 ^†^	231.9 ± 92.1	236.8 ± 106.2
C22:2[cis-13,16] 13,16-Docosadienoic acid	N/F		N/F	N/F	N/F
n-3					
C18:3[cis-9,12,15] alpha-Linolenic acid	10.3 ± 2.2		10.5 ± 3.4	11.9 ± 1.6	19.3 ± 6.2 ^†^
C20:3[cis-11,14,17] 11,14,17-Eicosatrienoic acid	N/F		N/F	N/F	N/F
C20:4[cis-5,8,11,14] Eicosa-5,8,11,14,17-pentaenoic acid	9.2 ± 2.4		10.7 ± 4.5	10.4 ± 1.7	16.0 ± 6.5
C22:6[cis-4,7,10,13,16,19] cis-4,7,10,13,16,19-Docosahexaenoic acid	39.1 ± 11.1		50.8 ± 14.1	33.7 ± 11.4	52.8 ± 28.0 *
n-6/n-3 PUFAs	14.9		12.5	13.1	9.3

Results are expressed as mean ± standard deviation (SD). n-3 PUFAs—n-3 polyunsaturated fatty acids; SFA—saturated fatty acids. <LOQ—below limit of quantification; N/F—not found. * *p* < 0.05 before vs. after within the group (Control or n-3 PUFA); † *p* < 0.05 difference between the groups.

**Table 6:** The effect of regular (Control group) and n-3 PUFAs enriched hen eggs (n-3 PUFAs group) consumption on serum pro- and anti-inflammatory cytokines, chemokines, vascular growth factors, and soluble cell adhesion molecules protein concentration.

Parameter	Control		n-3 PUFAs	
	Βefore	After	Βefore	After
IFNγ (pg/mL)	14.3 ± 8.7	16.6 ± 9.9	31.9 ± 31.9 †	12.2 ± 5.5
TNF-α (pg/mL)	11.1 ± 4.4	13.8 ± 6.1	9.1 ± 3.0	17.9 ± 16.4
IL-17A (pg/mL)	7.3 ± 4.8	9.7 ± 4.7 †	6.63 ± 2.57	9.2 ± 8.9
IL-6 (pg/mL)	32.4 ± 12.9	38.8 ± 17.1	32.6 ± 13.3	45.8 ± 28.5
IL-21 (pg/mL)	42.3 ± 30.1	46.8 ± 30.5	51.6 ± 48.2	80.7 ± 47.4
IL-22 (pg/mL)	31.6 ± 21.8	41.2 ± 34.3	36.5 ± 47.3	76.2 ± 59.2
IL-23 (pg/mL)	3.6 ± 0.5	3.7 ± 0.5	3.50 ± 0.31	4.0 ± 1.1
IL-9 (pg/mL)	4.9 ± 4.7	5.9 ± 5.2	4.0 ± 2.1	7.6 ± 11.7
IL-10 (pg/mL)	2.6 ± 1.1	3.2 ± 1.3 ^†^	2.7 ± 1.5	6.7 ± 4.6 *^,†^
SDF-1α (ng/mL)	1.0 ± 0.2	0.8 ± 0.3	1.3 ± 0.6	0.8 ± 0.4
LAP (ng/mL)	32.5 ± 16.8	27.4 ± 11.1	30.3 ± 11.8	29.4 ± 15.9
VEGF-A (pg/mL)	611.5 ± 480.9	684.3 ± 465.9 ^†^	527.8 ± 331.9	705.1 ± 602.6
VEGF-D (pg/mL)	0.4 ± 0.1	0.4 ± 0.1	0.5 ± 0.1 *	0.5 ± 0.3
sICAM-1 (ng/mL)	91.5 ± 51.2	95.2 ± 50.2	103.2 ± 46.5	107.0 ± 49.6
sVCAM-1 (ng/mL)	50.7 ± 17.7	48.2 ± 18.7	53.0 ± 23.1	55.1 ± 21.9

Data are presented as mean ± standard deviation (SD). n-3 PUFAs—n-3 polyunsaturated fatty acids; IFNγ—interferon gamma; TNF-α—tumor necrosis factor alpha; IL-17A—interleukin 17A; IL-6—interleukin 6; IL-21—interleukin 21; IL-22—interleukin 22; IL-23—interleukin 23; IL-9—interleukin 9; IL-10—interleukin 10; SDF-1α—stromal cell-derived factor 1 alpha; LAP—latency associated peptide; VEGF-A—vascular endothelial growth factor A ; VEGF-B—vascular endothelial growth factor D; sICAM-1—soluble intercellular adhesion molecule 1; sVCAM-1—soluble vascular cell adhesion molecule 1. * *p* < 0.05 difference between Control and n-3 PUFA group; † *p* < 0.05 difference between before and after within the group.

## Abbreviations

CV	cardiovascular
n-3 PUFAs	n-3 polyunsaturated fatty acids
BP	blood pressure
EPA	eicosapentaenoic acid
DHA	docosahexaenoic acid
ALA	alpha-linolenic acid
hsCRP	high-sensitivity C-reactive protein
NO	nitric oxide
ROS	reactive oxygen species
EPCs	endothelial progenitor cells
eNOS	endothelial nitric oxide synthase
iNOS	inducible nitric oxide synthase
BMI	body mass index
HR	heart rate
SBP	systolic blood pressure
DBP	diastolic blood pressure
MAP	Mean arterial pressure
FFM%	Fat Free Mass%
Fat%	Fat Mass%
TBW%	Total Body Water%
ECW%	Extracellular Water%
ICW%	Intracellular Water%
PF	Plasma Fluid
IF	Interstitial Fluid
HDL	high-density lipoprotein
LDL	low-density lipoprotein
GC–MS/MS	gas chromatography–tandem mass spectrometry
LDF	laser Doppler flowmetry
ACh	acetylcholine
AUC	area under the curve
SNP	sodium nitroprusside
INFγ	interferon gamma
TNF-α	tumor necrosis factor alpha
IL-17A	interleukin 17A
IL-6	Interleukin 6
IL-23	Interleukin 23
IL-9	Interleukin 9
IL-21	Interleukin 21
IL-22	Interleukin 22
IL-10	Interleukin 10
SDF-1α	stromal cell-derived factor 1 alpha
LAP	latency associated peptide
VEGF-A	vascular endothelial growth factor A
VEGF-D	vascular endothelial growth factor D
sICAM-1	soluble intercellular adhesion molecule 1
sVCAM-1	soluble vascular cell adhesion molecule 1
SD	Standard deviation
AChID	ACh-induced dilation
SNPID	SNP-induced dilation
